# Remarkable Response of Metastatic Gallbladder Carcinoma to Apatinib After Failed Multiline Chemotherapies: A Case Report and Literature Review

**DOI:** 10.3389/fonc.2019.01180

**Published:** 2019-11-08

**Authors:** Mengxi Zhang, Pengfei Zhang, Kexun Zhou, Qiu Li

**Affiliations:** ^1^Department of Medical Oncology, Cancer Center, West China Hospital, Sichuan University, Chengdu, China; ^2^West China Biomedical Big Data Center, Sichuan University, Chengdu, China

**Keywords:** metastatic gallbladder cancer, anti-angiogenesis, apatinib, chemotherapy, targeted therapy

## Abstract

Gallbladder carcinoma (GBC) is a relatively rare and aggressive malignant tumor with a poor prognosis. A systematic review of current clinical studies illustrates an extreme paucity of second-line therapeutic options following the failure of standard-of-care cisplatin-gemcitabine chemotherapy. The efficacy of apatinib, an highly potent and selective oral inhibitor of VEGFR-2 tyrosine kinase, for refractory advanced GBC has not yet been clarified. Herein, we report a case of advanced GBC that presented a durable partial response to apatinib used as monotherapy after the failure of multiline chemotherapies including S-1 monotherapy, capecitabine monotherapy, gemcitabine plus capecitabine, and irinotecan plus oxaliplatin. The patient achieved an efficacy of partial response within 2 months. By September 23, 2019, the duration of treatment had extended for almost 1 year with a satisfactory quality of life, and the administration of apatinib was continued. Dose reduction of apatinib occurred at week four due to grade 2 hypertension and hand-foot skin reaction (HFSR). No fatigue, proteinuria, mucositis, or thrombocytopenia occurred. To the best of our knowledge, this is the first case of a successful use of apatinib monotherapy for heavily pretreated GBC. Further prospective studies are warranted to confirm the efficacy and safety of apatinib in GBC.

## Background

Gallbladder carcinomas (GBC), relatively rare malignancies arising from epithelial cells of the gallbladder, account for 80–95% of all biliary tract cancers (BTC) and mainly present as mucin-producing adenocarcinomas (90% of patients) (1). Surgery is the only potentially curative treatment, and recurrence after resection remains common (2, 3). For unresectable, metastatic, or advanced BTC, the overall prognosis is still gloomy with a median overall survival (OS) of <12 months after the initial diagnosis; this is due mainly to the lack of effective second-line treatment.

Targeting the vascular endothelial growth factor (VEGF) pathway is a consolidated strategy in many cancer treatments. Apatinib is a small-molecule VEGF receptor 2 (VEGFR-2) tyrosinase inhibitor that has been demonstrated to have both an inhibitive effect on cell growth and to be anti-apoptotic.

Apatinib has been clinically proven to be safe and effective in treating advanced gastric cancer that failed to be treated with at least two lines of previous systemic therapy (4). In addition, apatinib exerts antitumor activities against a variety of tumor types, including breast cancer, non-small cell lung cancer, hepatocellular carcinoma, pancreatic cancer, and intrahepatic cholangiocarcinoma (5–9). However, the efficacy and safety of apatinib on cell migration and invasion in GBC are still indefinite.

Herein, we report a case of advanced GBC metastasized to the liver and lungs that presented a durable partial response (PR) to apatinib as monotherapy after the failure of multiline chemotherapies.

## Case Presentation

**Figure 1:**
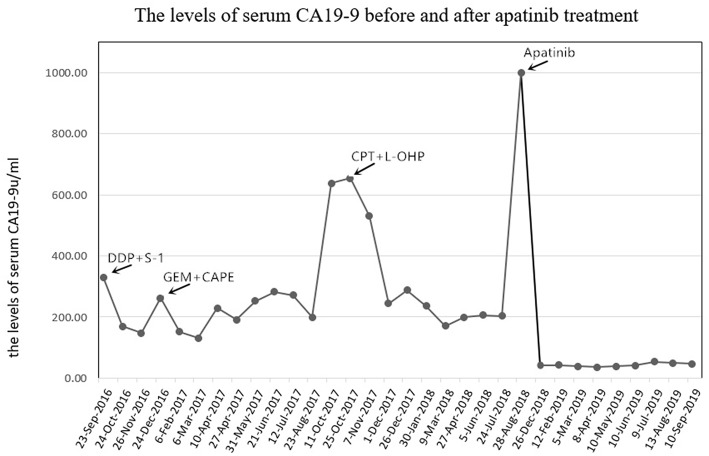
The levels of serum CA19-9 (normal range, 0–22 U/mL) before and after apatinib treatment. CA19-9, cancer antigen 19-9; DDP, cisplatin; GEM, gemcitabine; CAPE, capecitabine; CPT-11, irinotecan; L-OHP, oxaliplatin.

In January 2013, a 56-year-old female complained of persistent pain in the upper abdomen and back; this followed a reported 3 months of poor appetite, weight loss, and jaundice. T2-weighted magnetic resonance imaging (MRI) of the upper abdomen revealed gallbladder lesions. A surgical resection of the gallbladder was conducted on February 28, 2013, after which her jaundice was resolved. Histologically, the lesion was composed of intraductal papillary neoplasms with high-grade intraepithelial neoplasia together with some complex fusion and focal carcinoma. With regards to radical surgery, a margin negative resection status (R0-status) was reached and no positive lymph node was found. Thus, the patient was diagnosed with GBC at pT1aN0M0, Stage IA. No further chemotherapy or radiotherapy was given after surgery. On November 23, 2015, an MRI displayed a large mass occupying the gallbladder area and several soft tissue nodules in the lower segment of the common bile duct. The patient then received a palliative operation on December 17, 2015, including a total pancreectomy, splenectomy, subtotal gastrectomy, and partial hepatectomy. The pathology was confirmed as moderately differentiated adenocarcinoma. The preoperative serum CA19-9 was 731.30 U/mL (normal range, 0–22 U/mL) on November 24, 2015 (Figure 1), and then postoperative level decreased to 110.20 U/mL on March 23, 2016. During the 6-month follow-up period from March 2016 to September 2016, the laboratory tests demonstrated a durable increase in the serum CA19-9 level (330.50 U/mL on September 23, 2016). The patient was then administered three lines of chemotherapy regimens due to disease progression or serious adverse events, which included cisplatin plus S-1, gemcitabine plus capecitabine, and irinotecan plus oxaliplatin in sequence (Table 1). During administration of these chemotherapies, new disease progression in the liver and lungs was first confirmed by an upper-abdomen enhanced MRI and chest CT scan in October 2017.

**Table 1:** Summary of the timeline of the patient's medical history.

**Date**	**Treatment**	**Best efficacy**	**Adverse effects**
Sep. 2016–Jan.2017	DDP+ S-1	SD	Grade 2 nausea and hand–foot skin reaction
Jan. 2017–Dec.2017	GEM + CAPE	SD	Grade 1 nausea
Dec. 2017–Aug.2018	CPT-11 + L-OHP	SD	Grade 2 diarrhea
Sep. 2018–Jul.2019	Apatinib	PR	Grade 1 anorexia, fatigue; Grade 2 hypertension, hand–foot skin reaction, and diarrhea

*DDP, cisplatin; GEM, gemcitabine; CAPE, capecitabine; CPT-11, irinotecan; L-OHP, oxaliplatin; PD, progressive disease; PR, partial response; SD, stable disease*.

**Figure 2:**
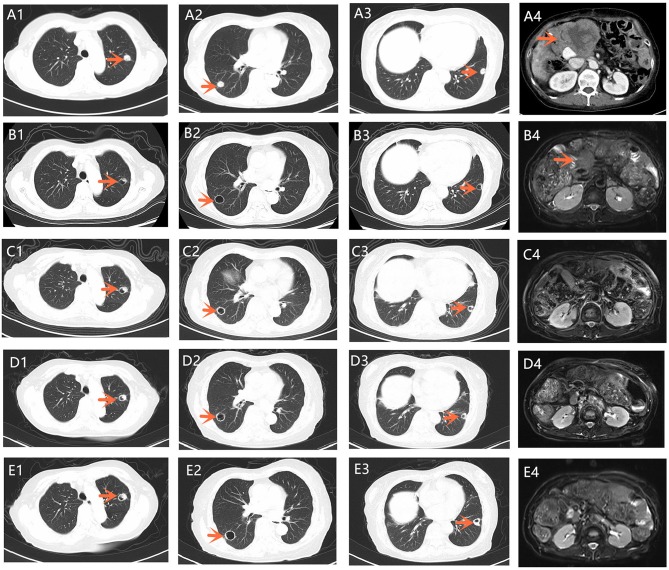
**(A1–A4)** A chest CT scan showed multiple metastases of both lungs. Total abdominal CT scan revealed a 7.8 × 6.9 cm mass located in the original gallbladder area, accompanied by abdominal and retroperitoneal lymph node metastasis (September 1, 2018); **(B1–B4)** A chest CT scan and T2-weighted abdominal MRI on November 16, 2018. The density of pulmonary metastases had considerably decreased with cavity formation compared with that in **(A1–A3)**. Tumor shrinkage was confirmed 2 months after apatinib was orally administered. **(C1–C4)** A chest CT scan and T2-weighted abdominal MRI on February 20, 2019. The mass located in the original gallbladder area reduced to the point of vanishing completely. **(D1–D4)** A chest CT scan and T2-weighted abdominal MRI on June 12, 2019. **(E1–E4)** A chest CT scan and T2-weighted abdominal MRI on September 16, 2019. CT, computed tomography; MRI, magnetic resonance imaging.

After seven cycles of the last chemotherapy regimen, the enhanced total abdominal CT scan on September 1, 2018, revealed a 7.8 × 6.9 cm mass located in the original gallbladder area (or the anastomotic stoma), accompanied by abdominal and retroperitoneal lymph node metastasis (Figures 2A1–A4). Meanwhile, the patient's serum CA19-9 level markedly elevated to >1000.00 U/ml. Accordingly, the efficacy was evaluated as systemic progression. After receiving written informed consent, we changed the regimen to apatinib at a dose of 500 mg qd in September 2018. Confirmed partial response (PR) was observed during CT and MRI re-examination within 2 months (November 16, 2018), with impressive reductions in both the numbers and size of the abdominal and retroperitoneal lymph node as well as the mass located in the anastomotic stoma, which shrunk to 5.2 × 3.1 cm (Figures 2B1–B4). Meanwhile, the density of the pulmonary metastases had considerably decreased with cavity formation. The levels of serum 19-9 significantly lowered to 40.60 U/ml. On February 20, 2019, the mass mentioned had reduced and vanished completely according to the diagnostic MRI (Figures 2C1–C4), and this was coupled with a normal level of serum 19-9. The patient experienced some side effects during treatment with apatinib, but no serious adverse events occurred. Anorexia (grade 1), fatigue (grade 1), and diarrhea (grade 2) were observed after 1 week of medication. Montmorillonite powder (3 g tid) was thus prescribed as indicated for the control and symptomatic relief of acute non-specific diarrhea. Hand–foot skin reaction and hypertension occurred after the first week of medication and aggravated to grade 2 after 2 weeks, which led to a 1-week suspension period of apatinib administration. The dose of apatinib was then reduced to 500 mg/d, and a 7-day treatment cycle of 5 days on followed by 2 days off was repeated. Hypertension normalized within 2 weeks following the administration of a combination of amlodipine (5 mg bid) and valsartan (40 mg bid). Meanwhile, the hand–foot skin reaction was also well-controlled after applying a fragrance-free cream that contains urea to keep hands and feet well-moisturized. No proteinuria, occult blood of feces, or decline in leucocyte count occurred. Although the levels of serum CA19-9 were slightly increased, the efficacy of PR was sustained for more than 10 months until the last reexamination (September 19, 2019), and this has since continued (Figures 2D1–D4,E1–E4). By the end of September 23, 2019, the administration of apatinib as single agent for maintenance therapy was continued and the duration of treatment reached almost 1 year with a satisfactory quality of life.

## Discussion

GBC, a malignancy usually associated with a history of gallstones, obesity, multiparity, and chronic infections (10), is the most prevalent BTC cancer type and has the worst prognosis. Although there are differences in the types of BTC (GBC, intrahepatic, and extrahepatic bile duct cancer), current systematic treatments tend to regard this group of tumors as a whole, which may complicate the assessment of treatment efficacy due to heterogeneous molecules and different clinical manifestations. Accordingly, these factors point to a growing need to individualize treatment for GBC patients. In accordance with the advent of the laparoscope and advanced imaging technology, there has been a sharp rise in the rate of early diagnosis and prompt operations for patients with GBC. However, recurrence following resection remains common.

In the last decade, progress has been made to identify the dose and scheduling of a combination of gemcitabine and cisplatin recommended for first-line standard treatment (11). The addition of nab-paclitaxel to gemcitabine-cisplatin has further prolonged PFS and OS (12). However, the prognosis of advanced BTC is still suboptimal with a median OS of only 12 months after initial diagnosis. Second-line treatments have thus far been primarily investigated alone or in combination in phase II trials. Some of these include cisplatin, S-1, capecitabine, irinotecan, oxaliplatin, and so on (13–18). More recently, in a prospective observational study, FOLFOX-4 as second-line treatment was evaluated to be an effective and well-tolerated regimen in locally advanced unresectable and metastatic GBC (19). However, the role of second-line treatment has not yet been determined, largely due to the prevalence of retrospective studies, the limited number of patients enrolled, and the heterogeneity of types explored.

Angiogenesis is one of the critical steps in tumor growth and hematogenous metastasis, and it plays a significant role in providing oxygen, nutrients, and growth factors to the tumor. The VEGFR2/RAF/MEK/ERK signaling pathway is involved in the process of angiogenesis and this pathway is frequently studied in a wide number of carcinomas, including GBC. In the last few years, some studies have indicated that VEGF-A is highly expressed in GBC, and that angiogentic microvessel density and the high expression of VEGF-A are correlated with poor prognosis of GBC, which suggests that GBC is actually angiogenesis-dependent (20–22). A literature review was conducted to identify the latest clinical trials exploring the medical efficacy of the anti-VEGF pathway in patients with metastatic BTC (Table 2). Eligible studies were identified through multiple databases (EMBASE, Web of science, PubMed, and the American society of clinical oncology (https://www.asco.org/) and the National Institutes of Health trial registry (Clinicaltrials.gov). Relevant studies were searched for using relevant medical terms for subject headings (“metastatic gallbladder carcinoma,” “advanced gallbladder carcinoma,” “biliary tract cancer,” “VEGFR,” “VEGF,” “apatinib,” “phase,” and “clinical trial”) and abstracts (“metast,” “VEGFR,” “VEGF,” “neoplasm metastasis,” “neoplasm recurrence,” and “biliary tract cancer”). The cut-off date for studies to be considered was August 24, 2019.

**Table 2:** Clinical trials targeting VEGF pathway for metastatic biliary tract cancer.

**Arm**	**Phase**	**Number**	**Main site of disease**	**TTP, mo**	**PFS, mo**	**OS, mo**	**ORR**	**DCR**	**References**
Regorafenib	II	43	IHCC: 62% EHCC: 26% GBC: 12%	–	15.6 weeks	31.8 weeks	11%	56%	(23)
Regorafenib + BSC vs. placebo + BSC	II	66	IHCC: 72.7% EHC: 15.2% GBC: 12.1%	–	3.0 vs. 1.5	–	–	70% vs. 33%	(24)
Sunitinib	II	56	IHCC: 62.5% EHCC: 10.7% GBC: 26.8%	1.7	–	4.8	8.9%	50%	(25)
Bevacizumab+ erlotinib	II	49		4.4	–	9.9	12%	63%	(26)
Bevacizumab + gemcitabine + oxaliplatin	II	35	IHCC: 62.9% EHCC: 8.5% GBC: 28.6%	–	7·0	12·7	40%	69%	(27)
Ramucirumab/Merestinib	II	ongoing	Advanced BTC	–	–	–	–	–	(28)
Lenvatinib	II	17	Unresectable BTC	–	–	–	5.9%	82%	(29)

*EHCC, extrahepatic cholangiocarcinoma; IHCC, intrahepatic cholangiocarcinoma; GBC, gall bladder carcinoma; BTC, biliary tract cancer; mo, month; TTP, time to progression; PFS, progression-free survival; OS, overall response rate; ORR, overall response rate; DCR, disease control rate*.

Regorafenib, sunitinib, and bevacizumab are currently the most frequently studied new-generation drugs that inhibit this specific signaling. Regorafenib is a multitargeted kinase inhibitor that inhibits VEGF signaling. A phase II trial (23) tested regorafenib as a single agent in 43 patients with chemotherapy refractory advanced BTC: PR was observed in five patients (11%), stable disease (SD) was achieved in 19 (44%). Median PFS was 15.6 weeks (90% confidence interval CI, 12.9–24.7) and OS was 31.8 weeks (90% CI, 13.3–74.3 weeks), with survival rates of 40% at 12 months and 32% at 18 months. Grade 3–4 toxicities were observed in 40% of patients and mainly presented as hypophosphatemia (40%), hyperbilirubinemia (26%), hypertension (23%), and hand-foot skin reaction (7%). Regorafenib was also evaluated in the phase II REACHIN trial (24), which suggested a significant increase in median PFS (3.0 vs. 1.5 months) and tumor control (70 vs. 33%), when compared with the placebo group, in patients with previously treated advanced BTC. Sunitinib, targeting VEGFRs, PDGFRs, and KIT, is also a potent anti-angiogenic tyrosine kinase inhibitor. In a phase II trial (25), sunitinib monotherapy demonstrated a marginal efficacy with an ORR of 8.9% and DCR of 50.0%. Similarly, the monoclonal antibody bevacizumab was used as an antiangiogenic agent combined with gemcitabine plus cisplatin or erlotinib and resulted in meaningful clinical outcomes in patients with advanced BTCs (26, 27). Despite small samples and various adverse events, these studies suggest that anti-vascular drugs might be promising for patients with advanced BTC. In addition, the anti-vascular targeting drugs ramucirumab (LY3009806) and lenvatinib, among others, are being investigated in phase II trials for use in treating previously treated advanced BTC (28, 29).

Apatinib is highly potent and a selective inhibitor targeting VEGFR-2 tyrosine kinase, and it is taken orally. With a high binding affinity to VEGFR2, apatinib significantly inhibits the migration and proliferation of endothelial cells, decreases tumor microvascular density, and promotes apoptosis. Based on the beneficial results from a randomized, double-blind, placebo-controlled phase III trial comparing apatinib and a placebo, apatinib was recommended as the third line to treat advanced gastric adenocarcinoma and gastroesophageal junction adenocarcinoma by the Food and Drug Administration of China in 2015. Until now, there has been only one retrospective analysis with a small sample size (21 patients were enrolled) that has evaluated the efficacy and safety of apatinib in advanced BTC (30). In this study, the primary cancer included 12 (57%) intrahepatic cholangiocarcinoma, 5 (24%) gallbladder, and 4 (19%) extrahepatic cholangiocarcinoma. Three patients (14%) (two were diagnosed with GBC) achieved PR and 12 (57%) achieved stable disease (SD), and the cancer control rate was 71%. Median PFS and OS were 2.8 months (95% CI 0.85–8.03) and 8.8 months (95% CI 2.85–14.7), respectively. Grade 3–4 adverse events were hypertension (14.2%), hand-foot syndrome (23.8%), proteinuria (9.5%), fatigue (9.5%), hyperbilirubinemia (4.7%), and diarrhea (4.7%).

In this case report, the patient was treated with apatinib monotherapy, and PR was observed within 2 months. Meanwhile, there was obvious cavity formation in the lung metastasis, which was consistent with the characteristics of the effect of anti-vascular targeted drugs (31). The efficacy of PR was sustained for at least 10 months with a satisfactory quality of life and this is still in extension, indicating that apatinib might be effective in heavily pretreated advanced GBC. In this case, the adverse events of apatinib mainly presented as anorexia, fatigue, diarrhea, hand–foot skin reaction, and high blood pressure. The adverse effects were controllable by dose reduction or interruption and symptomatic treatment.

In summary, we, for the first time, reported a successful case of apatinib with acceptable toxicity monotherapy in refractory advanced GBC after the failure of multiline chemotherapies. Targeting the VEGFR signaling pathway may be a beneficial strategy for GBC in the near future. Further prospective studies are warranted to confirm the efficacy and safety of anti-vascular therapy in GBC.

## Data Availability Statement

All datasets generated for this study are included in the article/supplementary material.

## Ethics Statement

The studies involving human participants were reviewed and approved by West China Hospital of Sichuan University Biomedical Research Ethics Committee. The patients/participants provided their written informed consent to participate in this study. Written informed consent was obtained from the individual(s) for the publication of any potentially identifiable images or data included in this article.

## Author Contributions

MZ performed the data acquisition. MZ and KZ performed the data analysis and interpretation. PZ performed the radiological analysis of MRI and CT images. MZ and QL performed the manuscript preparation.

### Conflict of Interest

The authors declare that the research was conducted in the absence of any commercial or financial relationships that could be construed as a potential conflict of interest.
